# Deepfake video detection: YOLO-Face convolution recurrent approach

**DOI:** 10.7717/peerj-cs.730

**Published:** 2021-09-21

**Authors:** Aya Ismail, Marwa Elpeltagy, Mervat Zaki, Kamal A. ElDahshan

**Affiliations:** 1 Mathematics Department, Tanta University, Tanta, Al-Gharbia, Egypt; 2 Systems and Computers Department, Al-Azhar University, Cairo, Nasr City, Egypt; 3 Mathematics Department, Al-Azhar University (Girls Branch), Cairo, Nasr City, Egypt; 4 Mathematics Department, Al-Azhar University, Cairo, Nasr City, Egypt

**Keywords:** Deepfake, YOLO-Face, Convolution recurrent neural networks, Deepfake detection, Video authenticity

## Abstract

Recently, the deepfake techniques for swapping faces have been spreading, allowing easy creation of hyper-realistic fake videos. Detecting the authenticity of a video has become increasingly critical because of the potential negative impact on the world. Here, a new project is introduced; You Only Look Once Convolution Recurrent Neural Networks (YOLO-CRNNs), to detect deepfake videos. The YOLO-Face detector detects face regions from each frame in the video, whereas a fine-tuned EfficientNet-B5 is used to extract the spatial features of these faces. These features are fed as a batch of input sequences into a Bidirectional Long Short-Term Memory (Bi-LSTM), to extract the temporal features. The new scheme is then evaluated on a new large-scale dataset; CelebDF-FaceForencics++ (c23), based on a combination of two popular datasets; FaceForencies++ (c23) and Celeb-DF. It achieves an Area Under the Receiver Operating Characteristic Curve (AUROC) 89.35% score, 89.38% accuracy, 83.15% recall, 85.55% precision, and 84.33% F1-measure for pasting data approach. The experimental analysis approves the superiority of the proposed method compared to the state-of-the-art methods.

## Introduction

Recent advancements in artificial intelligence, especially in deep learning, have facilitated generating realistic fake images and videos. The availability of open software Deepfake applications, like FakeApp and DeepFaceLab, has increased the tampered images and videos on social media and the Internet, leading to a public problem. The term “deepfake” consists of two stems; deep-learning and fake. Deepfake is a technique to put face photos of a donor person to an origin person in a certain video to generate a video of the donor saying or doing things said or done by the origin one. Deepfakes have several negative impacts on individuals, societies, and countries, since they can be misused to affect election results, cause political tension among countries, deceive individuals, and spread false news regarding celebrities, just to name a few; hence, detecting deepfakes is crucial.

Deepfake video detection is a classification problem where classifiers detect both genuine and tampered videos. Several detection methods have been presented after deepfakes were launched in 2017, most of which are based on deep learning. Manipulated video detection methods can be categorized into; methods based on detecting visual discrepancies and artifacts inside frames using deep convolutional neural networks, and methods based on detecting the temporal discrepancies across frames using deep recurrent neural networks (Nguyen et al., 2019b).

Since the process of generating deepfake videos has been expanding, there is a necessity to improve the existing face detection methods, and in turn, developing methods to detect deepfake videos with low loss and high accuracy which requires more effort. This paper proposes an efficient method which detects whether a video is genuine or fake. A modified version of YOLO-Face detector is employed to detect faces from video frames. Next, a fine-tuned convolution neural network (CNN)—such as EfficientNet-B5—is used to extract features of the detected faces. Then, all features extracted from videos are grouped as a batch of input sequences to pass into the recurrent neural network (RNN)—such as Bi-LSTM—to detect the temporal discrepancies. Finally, the probability of a video being either deepfake or genuine is computed. A merged challenge dataset – CelebDF-FaceForencics++ (c23)—is used to evaluate the robustness of the proposed scheme. This dataset is a combination of two popular datasets, which are FaceForencies++ (c23) and Celeb-DF.

In summary, this work presents the following contributions:

- A refined YOLO-Face detector version is presented to detect face areas from video frames in order to improve the performance of detecting the videos authenticity.
- A fine-tuned Convolution Recurrent Neural Network called EfficientNet-B5 Bi-LSTM is introduced to extract the spatial-temporal features from the short sequence of frames for detecting the videos authenticity. This is due to the fact that deepfake video was generated from processing facial synthesis frame-by-frame, and hence, pixels' values of video in synthesis regions are not coherent and consistent in spatial and temporal information.
- A combined CelebDF-FaceForencics++ (c23) dataset is introduced. It provides an integrated and diverse deepfake dataset, and helps to improve the applicability of the deepfake detection model in the real world.
- Comprehensive analysis of several deep-learning models applied in the context of deepfake detection is presented, in terms of AUROC, accuracy, recall, precision, and F-measure.

The rest of the work is organized as follows; “Related Work” reviews the deepfakes video creation techniques, current datasets, and deepfake detection methods. “The Proposed Method” introduces the proposed method for detecting face deepfakes in videos. The experimental result analysis is reported in “Experimental Result Analysis”. Finally, “Conclusion and Future Work” demonstrates the conclusion and future work.

## Related work

Various techniques for creating hyper-realistic deepfake faces have been recently launched every day. FakeApp was the first application created by a Reddit user for generating deepfakes using the autoencoder-decoder architecture (Güera & Delp, 2018). The autoencoder-decoder structure has been improved *via* adding two layers; adversarial loss and perceptual loss. Thus, an advanced version of deepfakes called Faceswap-GAN has been produced based on the Generative Adversarial Network (GAN) (Nguyen et al., 2019b). These layers are used to capture latent features of the face such as eye movements to generate more realistic deepfake images. GAN is composed of two artificial neural networks trained in tandem; a generator that creates data looking like the training data, and a discriminator that detects fake from real data (Géron, 2019).

In the deepfake detection problem, videos authenticity should be evaluated. This requires a large dataset to train the model. The most popular current deepfake video datasets are DeepFake-TIMIT (DF-TIMIT) (Korshunov & Marcel, 2018), UADFV (Yang, Li & Lyu, 2019), FaceForensics++ (Rossler et al., 2019), Google/Jigsaw DeepFake Detection (DFD) (Dufour & Gully, 2019), Celeb-DeepFake (Celeb-DF) (Li et al., 2020), Deepfake Detection Challenge (DFDC) (Dolhansky et al., 2019), and DeeperForensics-1.0 (Jiang et al., 2020) datasets. FaceForencics++ (FF++) is a large-scale dataset based on several different manipulation techniques: Face2Face, FaceSwap, Deepfakes, and NeuralTextures, for automatically creating fake faces in videos. That dataset was released early in the year 2019. It contains 1,000 original videos downloaded from the Youtube-8M dataset and 4,000 diverse manipulated videos. Face2Face and FaceSwap are computer graphics-based, while Deepfakes and NeuralTextures use a deep learning approach. FF++ dataset has been created in 3 different compression factors; raw, medium (c23), and high (c40). Celeb-DF dataset, released in 2019, consists of 590 original videos and 5,639 deepfake videos. It is generated using an improved DeepFake synthesis algorithm. It is considered more realistic than the previous datasets due to its manipulation process which produces few visual artifacts. The original videos in Celeb-DF dataset are selected from interviews on YouTube of 59 celebrities varying in genders, ethnic and ages groups. The average length of these videos is approximately 13 s with the frame rate 30 per second.

Various methods have been developed to detect videos deepfakes depending on either the inconsistencies in the temporal information or the visual artifacts and discrepancies within frames. Güera & Delp (2018) used the InceptionV3-LSTM temporal pipeline to detect the deepfakes on 600 videos collected from several websites and the HOHA dataset (Laptev et al., 2008). This method achieves an accuracy of 97%. In Li, Chang & Lyu (2018), the face areas are localized from video frames by the dlib face detector. Then, the facial landmarks are extracted, and the faces are aligned to a unified coordinate space using landmark-based face alignment algorithms. After that, the eye areas are detected based on eye landmarks and passed into the VGG16-LSTM model to learn the temporal patterns of eye blinking. This method is evaluated on 49 videos from the web and their corresponding deepfake videos created by a deepfake algorithm. Wubet (2020) employed VGG16 and ResNet-50 CNN models to extract features from eye frames, and to classify the eye states; opened or closed. Then, LSTM is applied to detect deepfakes on the UADFV dataset using eye blinking speed. This temporal method yields approximately an accuracy of 95%. Jiang et al. (2020) utilized the Inflated 3D ConvNet (I3D), and ResNet50-LSTM to extract the Spatio-temporal features and to detect the deepfakes on the DeeperForensics-1.0 dataset. The I3D model yields the best accuracy score of 79.25% on the hidden test set. Masi et al. (2020) aligned the face regions from video frames, and then two branches of DenseBlocks are employed to fuse the information data from the color and frequency domains. These DenseBlocks are followed by Bi-LSTM for temporal modeling to isolate the deepfakes. This method is trained on the FF++ dataset and evaluated on the Celeb-DF dataset where an AUROC score of 73.41% has been achieved.

Nguyen, Yamagishi & Echizen (2019) used a part of the VGG-19 CNN to extract the latent features from the detected and scaled face frames. These latent features are then fed as input to three capsule networks for deepfake detection. This method is evaluated on the deepfake dataset suggested by Afchar et al. (2018) which consists of online videos, and achieved an accuracy score of 99.23%. Nguyen et al. (2019a) designs a CNN-based autoencoder to detect the tampered videos and locate the tampered areas on the FaceForensics++ (c23) dataset. The face regions are extracted and fed as input to the autoencoder, which comprises an encoder and a Y-shaped decoder. This method yields an accuracy score of 52.32% for classification and 70.37% for segmentation. Li et al. (2020) used eight different CNN architectures to detect the deepfakes; namely: GoogLeNet, InceptionV3, Meso4, MesoInception4, ResNet-50, designed-CNN based on multilayer feed-forward network, XceptionNet, CapsuleNet based on VGG19, and Dual Spatial Pyramid (DPS) based on FWA. These CNNs are trained on different datasets and evaluated on the Celeb-DF dataset where the highest score 65.5% of an AUROC metric has been achieved using the XceptionNet-c40 model. Table 1 summarizes some of the video deepfake detection methods.

**Table 1:** Summary of some video deepfake detection methods.

Research paper	Face detector	Most efficient classifier	Datasets/Findings
Sabir et al. (2019)	Masks provided by Rossler et al. (2019)	DenseNet + Gated Recurrent Unit (GRU)	**FF++** dataset Accuracy: 96.9%
Singh et al. (2020)	MobileNet-SSD	EfficientNet-B1 + time-distributed layer + LSTM	**DFDC** dataset Accuracy: 97.6%
de Lima et al. (2020)	RetinaFace	3D CNNs	**Celeb-DF** dataset Accuracy: 98.26% AUROC: 99.73%
Montserrat et al. (2020)	MTCNN	EfficientNet-B5 + Automatic Face Weighting layer + GRU	**DFDC** dataset Accuracy: 91.88%
Afchar et al. (2018)	Viola-Jones	MesoInception-4	**Online** videos dataset Accuracy: 98%
Rossler et al. (2019)	Face tracking method	XceptionNet	**FF++** dataset Accuracy: 99.08%, 97.33%, and 86.69% for raw data, high quality data, and low-quality data, respectively
Li & Lyu (2018)	Dlib	ResNet50	**UADFV** dataset AUROC: 97.4% **Deepfake-TIMIT** dataset AUROC: 99.9% and 93.2% on low and high qualities, respectively
Dang et al. (2020)	InsightFace	XceptionNet + attention-based layer	Training dataset: **DFFD** Testing dataset: **UADFV** AUROC: 84.2% Testing dataset: **Celeb-DF** AUROC: 64.4%
Kumar, Bhavsar & Verma, 2020	MTCNN	XceptionNet	**Celeb-DF** dataset AUROC: 99.2%

## The proposed method

**Figure 1:**
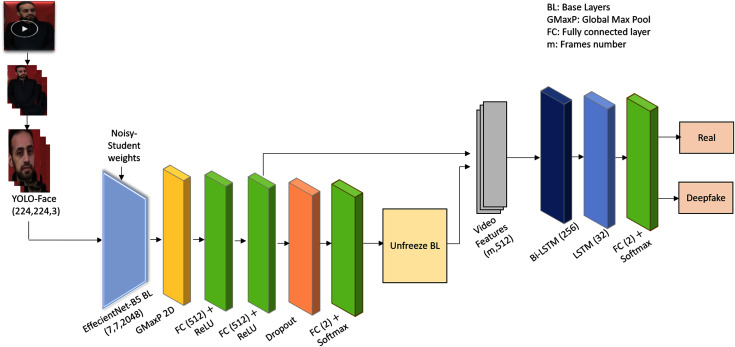
The YOLO-CRNNs deepfake detection system architecture.

The proposed framework presents an efficient deepfake video detection method. Figure 1 shows the system architecture of the proposed Deepfake detection method. As shown in Fig. 1, the proposed method detects faces using YOLO-Face detector, and then extracts the visual spatial-temporal features using CRNNs model. The CRNNs consist of pre-trained and fine-tuned convolution networks for spatial features extraction, recurrence networks for analyzing the temporal sequences to learn the inconsistency along the temporal domain, and a fully connected layer to detect both fake and original videos. A detailed description of the architecture is explained below.

***Step 1:* Pre-processing.** The video is converted into frames. Faces are of great significance in existing manipulation methods, and they occupy a small area of the video. Extracting features from the whole frame is not optimal, therefore, extracting features from the face area using a YOLO-Face detector should be the main task. The faces are detected out of frames using YOLO-Face detector.

You Only Look Once (YOLO) is a popular, and fast real-time object detection technique that uses only one neural network to take the entire image in a single shot. When YOLO gets an input image, it divides the image into S × S grid where S is a random value depending upon the grids’ size. Each grid cell predicts multiple bounding boxes containing an object, their confidence scores, and the class probabilities. The non-maximal suppression method is applied to the predicted boxes by removing the overlapping boxes and keeping only the necessary ones with a high probability to contain objects (Redmon et al., 2016). YOLOv3 is the third iteration of YOLO consisting of deep network architecture called darknet-53 which got impressive results on the COCO dataset (Redmon & Farhadi, 2018).

**Figure 2:**
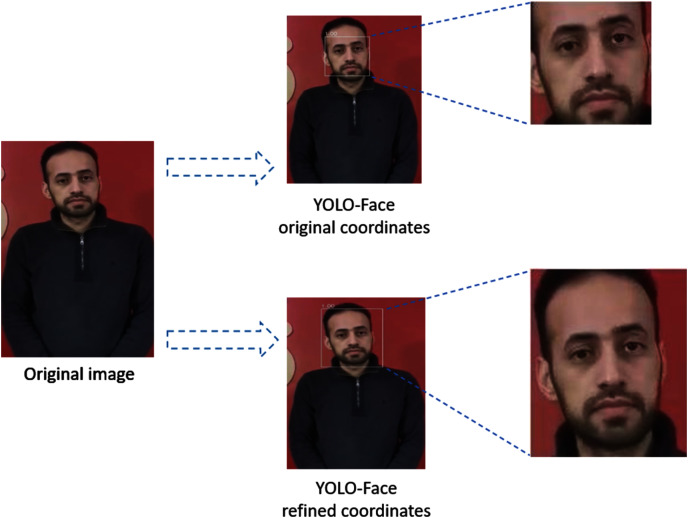
The difference between YOLO-Face refined coordinates and YOLO-Face original coordinates.

YOLO-Face is a face detection model resulting from improving the YOLOv3 architecture to predict the coordinates of faces and produce cropped faces using these coordinates (Chen et al., 2021). For deepfake detection, we refine the predicted coordinates, which are left, top, right, and bottom, of the YOLO-Face bounding box hence taking up a large area of the head that might hold artifacts, which is useful to detect deepfake in faces. This means that instead of using the YOLO-Face detector's original coordinates, which detect a small region of the face, we modify them by increasing the size of the detected bounding box by 22% proportional to its area to produce the whole face, as shown in Fig. 2. Then, the detected faces are resized into shape (224,224,3).

***Step 2:* Spatial features extraction.** The visual-spatial features for each face are extracted using one of the transfer-learned and fine-tuned deep pre-trained CNNs models; EfficientNet-B5. EfficientNet-B5 (Tan & Le, 2019) network is used after some modifications, such as excluding its top layer, as a base model and it has been pre-trained on Noisy-Student weights (Xie et al., 2020). This means that the final fully connected layer of the actual EfficientNet-B5 network, that transforms the features on its previous layer into 1,000 prediction classes of ImageNet, is excluded in order to add layers helping to detect videos authenticity. Afterwards, the base model is fine-tuned with a global maximum pooling layer for down sampling the feature maps of shape (7,7,2048) to a 2,048-neuron feature representation and passing the valid information from the previous layer. Next, two fully connected layers are added, in which every input is connected to every output by weight. A rectified linear activation function (ReLU) is used to add nonlinearity allowing to learn the complex relationships in the data. A dropout layer (Srivastava et al., 2014) is added as a regulator to prevent overfitting during training and to enhance the generalization ability of the model. In addition, a fully connected layer is added as an output layer. As the ImageNet dataset contains 1,000 various classes of images, the base model is re-trained with face data to force the first layers to focus on the facial rather than generic features.

***Step 3:* Temporal features extraction and classification.** Deepfake videos lack temporal consistency as frame-by-frame video forgery generates artificial low-level features that appear as incoherent temporal artifacts. To exploit this weak spot, the visual spatial features for each frame in the video are fed as a batch of input sequences to recurrence network, such as Bi-LSTM, to extract the temporal features. LSTM (Graves, Fernández & Schmidhuber, 2005) is an extension of RNN architecture that was created to learn chronological dependence in long-range sequence prediction issues, and Bi-LSTM is considered as an upgrade of LSTM, in which the training sequence proceeds forward and backward. Bi-LSTM with 256 hidden units is used followed by LSTM with 32 units as recurrence networks to learn the temporal discrepancies among video frames. LSTM is followed by a fully connected layer as an output prediction layer with two units representing the number of classes and a Softmax activation function for detecting real and deepfake videos. The proposed method’s layers are shown in Table 2.

**Table 2:** Description of the proposed method’s layers.

Layer (type)	Output shape	Parameters #
efficientnet-b5 (Model)	(None, 7, 7, 2048)	28,513,520
global_max_pooling2d (GlobalMax)	(None, 2048)	0
dense (Dense)	(None, 512)	1,049,088
dense_1 (Dense)	(None, 512)	262,656
dropout_1 (Dropout)	(None, 512)	0
dense_2 (Dense)	(None, 2)	1,026
main_input (InputLayer)	[(None, 10, 512)]	0
bidirectional (Bidirectional (LSTM))	(None, 10, 512)	1,574,912
lstm_1 (LSTM)	(None, 32)	69,760
Dense_3 (Dense)	(None, 2)	66
Total parameters: 31,471,028 Trainable parameters: 31,298,292 Non-trainable parameters: 172,736		

### Dataset description

A combined challenge dataset – CelebDF-FaceForencics++ (c23)—has been used to evaluate the robustness of the proposed scheme. This dataset consists of merging two datasets, which are Celeb-DF and FF++ (c23). Table 3 shows the actual number of real and fake videos in both datasets. The Celeb-DF dataset is originally divided into 5,299/712 for training data and 340/178 for testing data as fake and real videos.

**Table 3:** Real and fake video numbers of Celeb-DF and FF++ (c23) original datasets.

Dataset	Real number	Deepfake number
Celeb-DF	590 (Celeb-real) + 300 (YouTube-real)	5639 (deepfake videos of Celeb-fake)
FF++ (c23)	1,000	1,000

To train the proposed method, the above-mentioned training Celeb-DF videos are used with random sample real and deepfake video sets, each of length 712, from the FF++ (c23) dataset. To overcome the imbalance between fake and original classes, two approaches are applied to Celeb fake class before the merge process. The first one is called bootstrap aggregating which is a machine learning ensemble algorithm that repeatedly produces several training samples drawn with replacement (Breiman, 1996). The other one is called pasting which generates different numbers of training samples drawn without replacement (Re & Valentini, 2012). Both approaches are used to reduce the training data overfitting to create strong learners for producing accurate predictions. The final classification result for the models that used these approaches is recorded by voting. Therefore, the first approach is used to select “n” number of random samples with replacement, each of length 712 from the fake Celeb videos, while the second approach is used to divide the Celeb fake videos to 7 $\left( {5299/712 \approx 7} \right)$ different equal number of random samples without replacement each of length 712. The details for constituting the training CelebDF-FaceForencies++ (c23) sets for pasting and bootstrap aggregating approaches are shown in Table 4. To test the proposed method, 518 real and deepfake videos of the above-mentioned testing Celeb-DF dataset are used to simulate the real-world scenario as shown in Table 4. The Celeb-DF set is specially selected as a test set because its deepfake videos have high visual quality. This produces hyper-realistic videos similar to the real-world scenes.

**Table 4:** Real and deepfake numbers for training and testing distributions of the CelebDF-FaceForencics++ (c23) dataset in pasting and bootstrap aggregating data approaches.

Data approach	Training data	Testing data
Pasting	7*2,848 = 7* (712 Celeb-real+712 Celeb-fake (totally different videos for each random selection) +712 FF++(c23)-real+712 FF++(c23)-fake)	518 = (340 Celeb-fake+178 Celeb-real)
Bootstrap aggregating	2,848 = (712 Celeb-real+712 Celeb-fake (using random selection) +712 FF++(c23)-real+712 FF++(c23)-fake)	

## Experimental result analysis

In this section, we analyze the effectiveness of the suggested scheme in the light of conducted experiments. The proposed method of deepfake video detection is trained by both the training set of CelebDF-FaceForencies++ (c23) dataset and the training set of FF++ (c23) dataset, and the evaluation is performed by using the test set of Celeb-DF dataset. The training sets are split into random train and validation subsets. The image pixels are normalized into range (−1,1).

### Performance measures

A well-known evaluation metric, called an Area Under the Receiver Operating Characteristic (AUROC) curve, is used to evaluate the usefulness and productivity of the proposed deepfake detection method. AUROC curve is considered one of the most important measurements for evaluating the performance of classification models. It represents the ability of the model to discriminate between positive and negative examples. It is defined as the region enclosed by the coordinate axis under the Receiver Operating Characteristic (ROC) curve. The ROC curve is produced by plotting true positive (TP) rate on *Y*-axis and false positive (FP) rate on *X*-axis for a various number of threshold values (Fawcett, 2006). TP and FP rates can be computed as follow:

$${\rm TP\; rate} = \displaystyle{{true\; positives\; number} \over {true\; positives\; number + false\; negatives\; number}}$$

$${\rm FP\; rate} = \displaystyle{{false\; positives\; number} \over {false\; positives\; number + true\; negatives\; number}}$$

The higher the AUROC, the better the model performance is at distinguishing between real and deepfake videos. In addition, accuracy is another evaluation metric in which the detection model is assessed by considering the ratio of correctly predicted samples to the total number of predicted samples. Furthermore, to confirm the detection results, recall, precision, F1-measure, and confusion matrix are also used to assess the performance of the proposed model. The mathematical formulae of accuracy, recall, precision, and F1-measure are calculated based on true positive, false negative, false positive, and true negative samples’ numbers on the test set as follows.

$${\rm accuracy} = \displaystyle{{true\; positives\; number + true\; negatives\; number} \over {total\; samples\; number}}$$

$${\rm recall} = \displaystyle{{true\; positives\; number} \over {true\; positives\; number + false\; negatives\; number}}$$

$${\rm precision} = \displaystyle{{true\; positives\; number} \over {true\; positives\; number + false\; positives\; number}}$$

$$\text{F{‐}measure} = \displaystyle{{2 \times {\rm precision\; } \times recall} \over {{\rm precision} + {\rm recall}}}$$

### Experimental results and analysis

To justify the selection of the proposed scheme building blocks and ensure its robustness, many experiments have been conducted as follows.

***Experiment 1:*** In this experiment, the selection of face detector is justified by comparing it with the popular state-of-the-art detector; MTCNN (Zhang et al., 2016). The YOLO and MTCNN face detectors are applied to the CelebDF-FaceForencies++ (c23) dataset. The proposed YOLO based method achieves better performance than MTCNN based methods as shown in Table 2, since the YOLO detector produces a fewer number of false positives compared to the MTCNN. The YOLO based method achieved 89.35% of AUROC score for pasting approach and 85.12% for bootstrap aggregating approach. The MTCNN based method produced 83.39% and 82.87%, respectively. Meanwhile, as shown in Table 5, the AUROC results for pasting approach on the new merged dataset exceeded that for bootstrap aggregating approach, since pasting was originally designed for large datasets (Breiman, 1999).

**Table 5:** Comparative study of the proposed method with the other state-of-the-art methods on the new CelebDF-FaceForencies++ (c23) training sets and the Celeb-DF testing set.

Method	AUROC test result (%)	Data approach
The proposed method	89.35	Pasting
YOLO EfficientNet-B5 (Step 1 and Step 2 of the proposed method) + [LSTM (Güera & Delp, 2018)]	84.88	Pasting
YOLO EfficientNet-B0 + [Bi-LSTM (Masi et al., 2020)]	81.32	Pasting
YOLO EfficientNet-B0 + [LSTM (Güera & Delp, 2018)]	80.98	Pasting
YOLO + [XceptionNet (Rossler et al., 2019)] + [Bi-LSTM (Masi et al., 2020)]	79.97	Pasting
YOLO + [XceptionNet (Rossler et al., 2019)] + [LSTM (Güera & Delp, 2018)]	79.40	Pasting
[MTCNN EfficientNet-B5 (Montserrat et al., 2020)] + [Bi-LSTM (Masi et al., 2020)]	83.39	Pasting
[MTCNN EfficientNet-B5 (Montserrat et al., 2020)] + [LSTM (Güera & Delp, 2018)]	80.41	Pasting
The proposed method	85.12	Bootstrap aggregating
[MTCNN EfficientNet-B5 (Montserrat et al., 2020)] + [ Bi-LSTM (Masi et al., 2020)]	82.87	Bootstrap aggregating

***Experiment 2:*** In this experiment, the selection of CNN model is justified by comparing it with other state-of-the-art models. Various architectures of CNN such as EfficientNet-B5, EfficientNet-B0 (Tan & Le, 2019) and XceptionNet (Chollet, 2017) are used as base models that were pre-trained either using Noisy-Student or ImageNet weights. They are applied on the CelebDF-FaceForencies++ (c23) dataset after the following modifications. Some layers are added such as global max pool 2D followed by two fully connected layer with 512 units and ReLU activation function. Next, a dropout layer is added to drop some units from input with a 0.2 probability rate for both EfficientNet-B5 and XceptionNet, and a 0.5 rate for EfficientNet-B0. The dropout layer is followed by a fully connected layer with two units and Softmax activation function. Additionally, these base models are unfrozen to focus on learning face features. As shown in Table 5, the EfficientNet-B5 based method outperforms the other CNN based methods by 8–10% of AUROC score. EfficientNet network that pre-trained on Noisy-Student weights is more powerful than that pre-trained on ImageNet weights, and the proposed method recorded the highest performance in the table.

***Experiment 3:*** In this experiment, two scenarios are carried out. The first one employs Bi-LSTM with 256 hidden units followed by LSTM with 32 units. The other one employs LSTM with 32 hidden units, to learn the sequences. Finally, each scenario is followed by a fully connected layer as an output prediction layer, with two units representing the number of classes and Softmax activation function, for detecting real and deepfake videos. The Bi-LSTM based methods attain higher performance than the LSTM based methods, as shown in Table 5. Whilst the YOLO EfficientNet-b5 Bi-LSTM registers an AUROC score of 89.35%, the YOLO EfficientNet-b5 LSTM records 84.88%.

***Experiment 4:*** When the training dataset shares a similar distribution to the test set, the applicability of the model may decrease in real-world detection scenarios. Therefore, in this experiment, the proposed method is trained by FF++ (c23) dataset while the evaluation is performed using Celeb-DF test set. 2,000 original and deepfake videos are used for training from FF++ (c23) dataset, while 518 real and fake videos are used for testing from Celeb-DF dataset. As shown in Table 6, the YOLO based method for FF++ (c23) dataset records 77.41% outperforming the other MTCNN based methods and the work presented in Masi et al. (2020) based on aligned frames. Additionally, the AUROC score of YOLO EfficientNet-b5 Bi-LSTM reaches 77.41%, while that for LSTM declines by 2.1%.

**Table 6:** Comparative study of the proposed detection method with the other state-of-the-art methods on FF++ (c23) training set and Celeb-DF testing set.

No.	Method	AUROC test result (%)	McNemar’s pairwise statistical test comparison with the proposed method
1	The proposed method	77.41	–
2	YOLO EfficientNet-B5 (Step 1 and Step 2 of the proposed method) + [LSTM (Güera & Delp, 2018)]	75.31	9.0
3	[MTCNN EfficientNet-B5 (Montserrat et al., 2020)] + [Bi-LSTM (Masi et al., 2020)]	73.66	10.6
4	[MTCNN EfficientNet-B5 (Montserrat et al., 2020) ] + [LSTM (Güera & Delp, 2018)]	70.29	13.5
5	Aligned-Frames Dense-Blocks Bi-LSTM (Masi et al., 2020)	73.41	9.8

Moreover, AUROC based comparative studies between the proposed method and the state-of-the-art methods are presented in Tables 5 and 6. As shown in Tables 5 and 6, the AUROC score on the Celeb-DF test dataset is high when training the methods on the new merged CelebDF-FaceForencies++ (c23) dataset compared to the FF++ (c23) dataset. This is due to the fact that instead of using a single dataset, merging two datasets makes the new one more diversifiable to resemble videos that may be encountered in the real world. This plays an important role in improving the evaluation results of the proposed deepfake detection method on the Celeb-DF test set that has high-quality videos resembling the real-world ones.

Furthermore, McNemar’s test has been employed to analyze the statistical significance of the proposed method performance compared to the other methods performances presented in Table 6. McNemar’s test is based on a chi-square $({\chi ^2})\;$distribution. It is applied to a $2 \times 2$ contingency table in which the cells include the number of instances correctly and incorrectly classified by both methods and the number of instances only identified correctly by one method (Samui, Roy & Balas, 2017). The test statistic is calculated from the following formula with 1 degree of freedom:

$${{\rm {\rm X}}^2} = \displaystyle{{{{\left( {{b_{ik}} - {c_{ki}}} \right)}^2}} \over {{b_{ik}} + {c_{ki}}}}$$

where ${b_{ik}}$ represents the number of instances misclassified by method $i$ but identified correctly by method $k$, and ${c_{ki}}$ represents the number of instances misclassified by method $k$ but not by method $i$. If the estimated test value is greater than the chi-squared table value of 3.84 at a 95% confidence interval, then the difference of the two classification methods results is statistically significant. Again, Table 6 shows McNemar’s test comparison between the proposed method and the other state-of-the-art methods on the FF++ (c23) training dataset and the Celeb-DF testing dataset. As can be seen from Table 6, McNemar’s statistical test confirmed that differences in classification result success are statistically significant for every pairwise comparison between the proposed method and the other state-of-the-art methods.

Since deep learning is usually used to reduce the difference between the target and predicted outputs, called a Loss function, we aim to minimize this loss function by finding the optimal weight values. Therefore, the Nesterov-accelerated Adaptive Moment Estimation (Nadam) optimizer (Dozat, 2016) is used to update the weight parameters to reduce the loss function. This optimizer is employed with learning rate 0.00002 and schedule decay 0.00004. Learning rate is defined as the size of an update to the model during each step and is considered as the most significant hyperparameter to tune for achieving good performance. Since decaying the learning rate diminishes overfitting in training data and achieves higher classification accuracy, schedule decay is used to adjust the learning rate during training by reducing its value depending on a pre-defined value. Moreover, as the cross-entropy loss plays a vital role in the training process of network, it is used as a loss function on the proposed model to measure whether this model is good enough or not, by estimating the difference between actual and predicted probability distributions (Zhu et al., 2020). Furthermore, Model-Check-Point is used to save the weights of model achieving the best performance on validation loss quantity. Early-stopping is used to stop training when the validation loss metric stops improving for a certain number of epochs.

**Figure 3:**
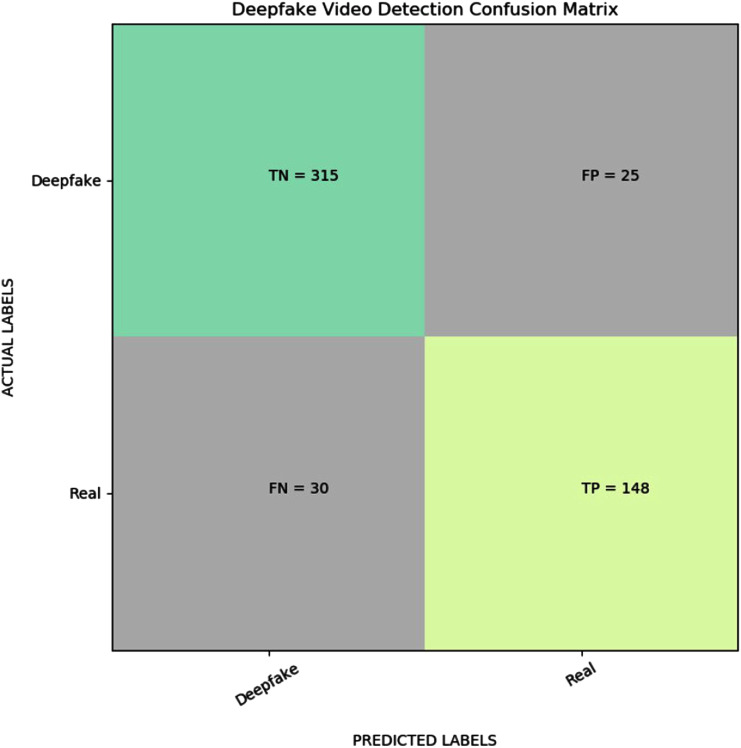
Visualization of confusion matrix for the proposed deepfake video detection method.

**Figure 4:**
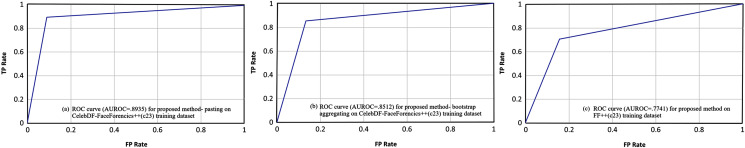
The AUROC curves for the proposed method on the CelebDF-FaceForencies++ (c23) and FF++(c23) dataset.

**Figure 5:**
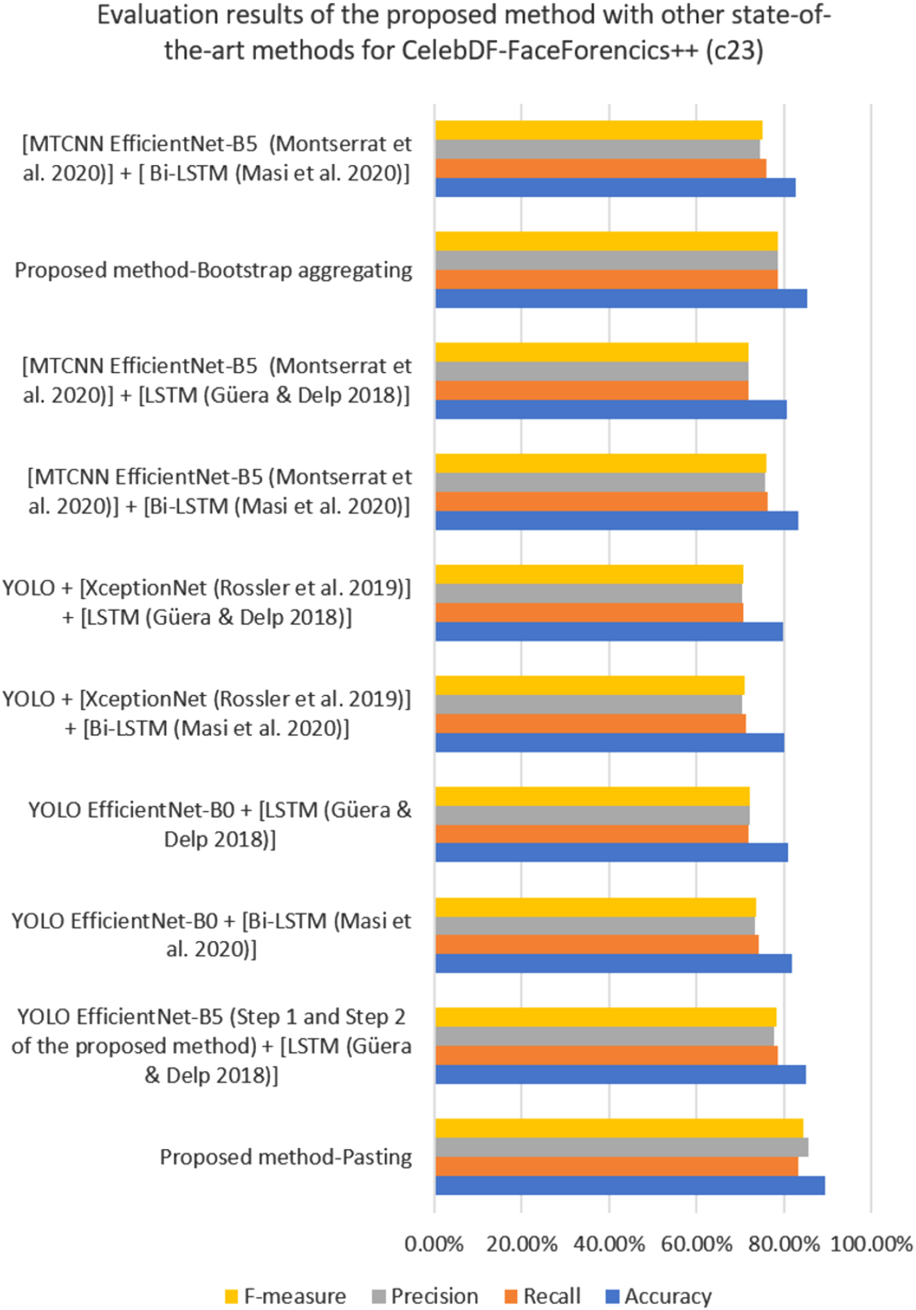
The performance of the proposed deepfake detection method compared to the other detection methods on the CelebDF-FaceForencies++ (c23) dataset.

**Figure 6:**
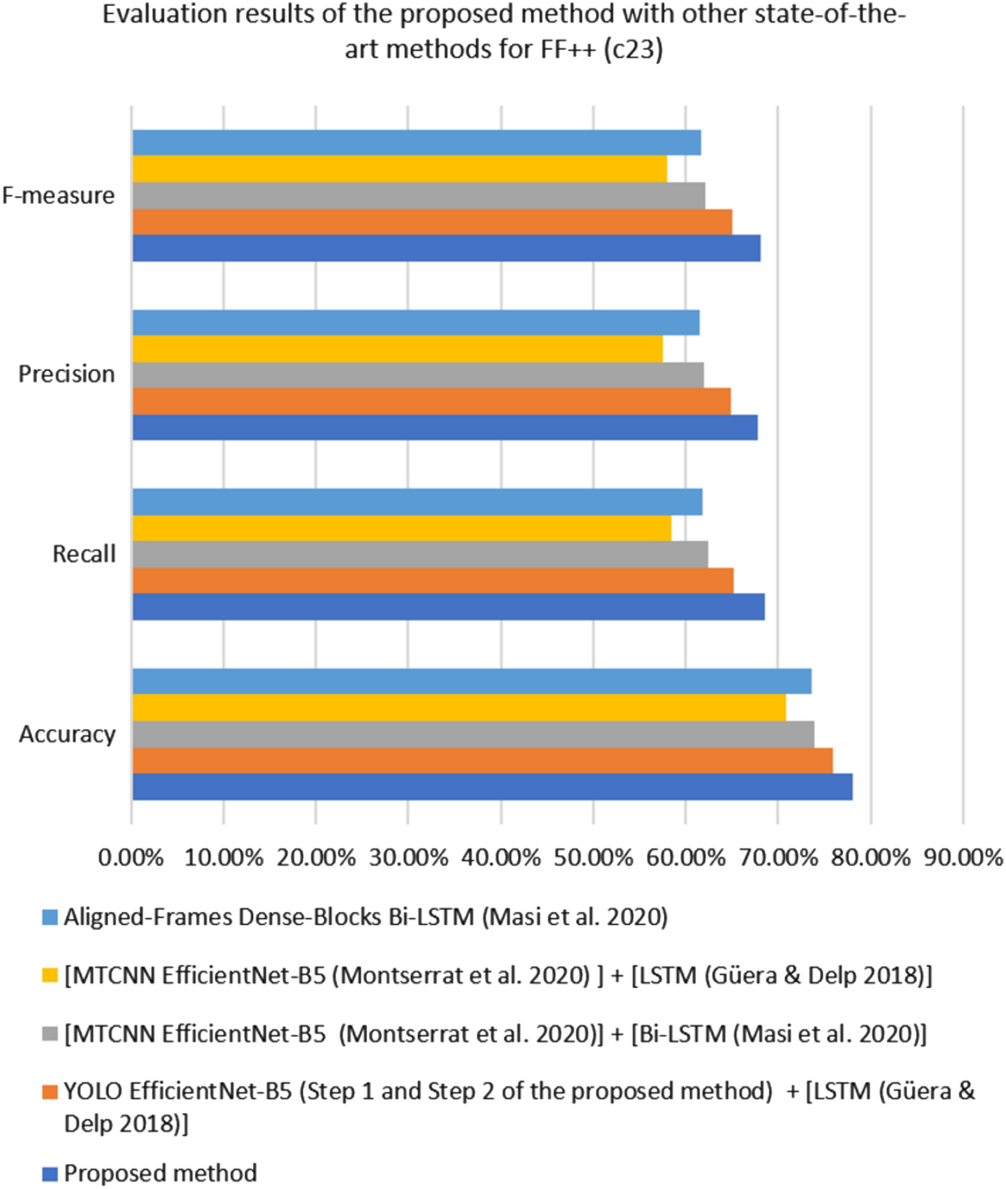
The performance of the proposed method compared to the other detection methods on the FF++ (c23) dataset.

Figure 3 shows the confusion matrix result for the proposed YOLO EfficientNet-b5 Bi-LSTM method based on the pasting approach to detect deepfake in videos. As can be shown from Table 7, the proposed method achieves 89.38% of accuracy, 83.15% of recall, 85.55% of precision, and 84.33% of F1-measure, respectively. In addition, Fig. 4 shows the AUROC curves corresponding to the performance of the proposed method for FF++(c23) dataset, and pasting and bootstrap aggregating approaches on the merged CelebDF-FaceForencies++ (c23) dataset. From Fig. 4A, it is clear that the ROC curve is close to the upper-left corner which assures a high performance by the suggested method for pasting approach on the CelebDF-FaceForencies++ (c23) dataset. Figures 5 and 6 display the evaluation results of comparing the proposed method to state-of-the-art methods for training the CelebDF-FaceForencies++ (c23) and FF++ (c23) datasets, and testing on the Celeb-DF test set. The previous experiments and evaluation results show that the proposed method outperforms the other state-of-the-art methods.

**Table 7:** Performance of the proposed method based on pasting data approach.

	Accuracy	Recall	Precision	F1-Measure
YOLO EfficientNet-B5 Bi-LSTM	0.8938	0.8315	0.8555	0.8433

The experiments have been run on a laptop equipped with RTX 2060 GPU-6 GB and Intel (R) Core (TM) i7-9750H CPU-16 GB on Windows 10. The code for deepfake detection proposed method is implemented using python programming language, especially version 3.7.4. Tensorflow, Keras, OpenCV, Sklearn, Numpy, OS, PIL, Random, Facenet_pytorch and Matplotlib are some of the python libraries used for achieving the proposed scheme.

## Conclusion and future work

In this paper, a new method for deepfake video detection is presented. The proposed method employs the YOLO-Face detector to detect face regions in video frames. The fine-tuned EfficientNet-B5 is employed to extract the spatial features of these faces, while the Bi-LSTM is employed to extract the temporal features across a video. A new large-scale dataset; CelebDF-FaceForencics++ (c23); based on merging two popular datasets; FaceForencies++ (c23) and Celeb-DF, is introduced. The proposed method achieves a high deepfake detection score using AUROC, accuracy, recall, precision, and F-measure metrics. An AUROC 89.35% score, 89.38% accuracy, 83.15% recall, 85.55% precision, and 84.33% F1-measure are recorded for the proposed method based on pasting approach. Comparative analyses revealed that the suggested method outperforms the state-of-the-art methods.

Since the techniques for producing deepfake videos progress continuously, we need to keep up by improving the current detection methods using different architectures and various face detection methods. Moreover, this work focuses on detecting deepfakes from faces in videos and ignores the audio content which could present an important improvement for the accuracy of deepfake detection methods in future work. Since merging YOLO with XGBoost was shown a good performance in object detection (Dave et al., 2021), this combination may be used to ameliorate the proposed method performance.

## Supplemental Information

10.7717/peerj-cs.730/supp-1 Supplemental Information 1 Extract video frames.

10.7717/peerj-cs.730/supp-2 Supplemental Information 2 Extract yolo faces part 1.

10.7717/peerj-cs.730/supp-3 Supplemental Information 3 Extract yolo faces part 2.

10.7717/peerj-cs.730/supp-4 Supplemental Information 4 Prepare data using pasting approach.

10.7717/peerj-cs.730/supp-5 Supplemental Information 5 Prepare data using bootstrap aggregating approach.

10.7717/peerj-cs.730/supp-6 Supplemental Information 6 Train data using efficientNet-b5.

10.7717/peerj-cs.730/supp-7 Supplemental Information 7 Extract video features for Bidirection-LSTM.

10.7717/peerj-cs.730/supp-8 Supplemental Information 8 Train the temporal model Bidirectional-LSTM.

10.7717/peerj-cs.730/supp-9 Supplemental Information 9 Test on 518 test set.

10.7717/peerj-cs.730/supp-10 Supplemental Information 10 Code Steps. 1. extract-video-frames.py: Extract video frames
2. yoloface.py, utils.py: Extract Yolo faces part 1, part 2
3. prepareData-Pasting.py: Prepare data (CelebDF-FaceForencics++ (c23)) using pasting approach
4. prepareData-Bootstrap aggregating.py: Prepare data (CelebDF-FaceForencics++ (c23)) using bootstrap aggregating approach
5. Train.py: Train data using EfficientNet-b5 (pre-trained on noisy student weights) on each training set for spatial features
6. BidirectionLSTM-Features.py: Extract video features for Bidirectional-LSTM
7. Train-Conv-Bi-LSTM.py: Train the temporal model Bidirectional-LSTM to learn the sequences
8. evaluate-model.py: Test on 518 test set of CelebDF dataset

## Additional Information and Declarations

### Data Availability

The following information was supplied regarding data availability: The FaceForencies++ dataset is available from the FaceForensics site: https://github.com/ondyari/FaceForensics. Rossler A, Cozzolino D, Verdoliva L, Riess C, Thies J, and Nießner M. 2019. Faceforensics++: Learning to detect manipulated facial images. Proceedings of the IEEE/CVF International Conference on Computer Vision. pp. 1–11. The Celeb-DF dataset is available from the celeb-deepfakeforensics site: https://github.com/yuezunli/celeb-deepfakeforensics. Li Y, Yang X, Sun P, Qi H, and Lyu S. 2020. Celeb-df: A large-scale challenging dataset for deepfake forensics. Proceedings of the IEEE/CVF Conference on Computer Vision and Pattern Recognition. pp. 3207–3216. The Python scripts are available in the Supplemental Files.
